# Ceftriaxone therapy attenuates brain trauma in rats by affecting glutamate transporters and neuroinflammation and not by its antibacterial effects

**DOI:** 10.1186/s12868-021-00659-8

**Published:** 2021-09-14

**Authors:** Sher-Wei Lim, Hui-Chen Su, Tee-Tau Eric Nyam, Chung-Ching Chio, Jinn-Rung Kuo, Che-Chuan Wang

**Affiliations:** 1 Department of Neurosurgery, Chi-Mei Medical Center, Chiali, Tainan, Taiwan; 2 Departments of Pharmacy, Chi-Mei Medical Center, Tainan, Taiwan; 3 Departments of Neurosurgery, Chi-Mei Medical Center, 901 Chung Hwa Road, Yung Kang City, Tainan, Taiwan; 4 Departments of Medical Research, Chi-Mei Medical Center, Tainan, Taiwan; 5 Department of Nursing, Min-Hwei College of Health Care Management, Tainan, Taiwan; 6 Center for General Education, Southern Taiwan University of Science and Technology, Tainan, Taiwan

**Keywords:** Traumatic brain injury, Ceftriaxone, Glutamate transporter, Glutamate receptor Neuroinflammation, Apoptosis

## Abstract

**Background:**

Ceftriaxone is a β-lactam antibiotic used to treat central nervous system infections. Whether the neuroprotective effects of ceftriaxone after TBI are mediated by attenuating neuroinflammation but not its antibacterial actions is not well established.

**Methods:**

Anesthetized male Sprague–Dawley rats were divided into sham-operated, TBI + vehicle, and TBI + ceftriaxone groups. Ceftriaxone was intraperitoneally injected at 0, 24, and 48 h with 50 or 250 mg/kg/day after TBI. During the first 120 min after TBI, we continuously measured heart rate, arterial pressure, intracranial pressure (ICP), and cerebral perfusion pressure. The infarct volume was measured by TTC staining. Motor function was measured using the inclined plane. Glutamate transporter 1 (GLT-1), neuronal apoptosis and TNF-α expression in the perilesioned cortex were investigated using an immunofluorescence assay. Bacterial evaluation was performed by Brown and Brenn’s Gram staining. These parameters above were measured at 72 h after TBI.

**Results:**

Compared with the TBI + vehicle group, the TBI + ceftriaxone 250 mg/kg group showed significantly lower ICP, improved motor dysfunction, reduced body weight loss, decreased infarct volume and neuronal apoptosis, decreased TBI-induced microglial activation and TNF-α expression in microglia, and increased GLT-1 expression in neurons and microglia. However, the grades of histopathological changes of antibacterial effects are zero.

**Conclusions:**

The intraperitoneal injection of ceftriaxone with 250 mg/kg/day for three days may attenuate TBI by increasing GLT-1 expression and reducing neuroinflammation and neuronal apoptosis, thereby resulting in an improvement in functional outcomes, and this neuroprotective effect is not related to its antibacterial effects.

## Introduction

After traumatic brain injury (TBI), excitotoxicity [1] and neuroinflammation [2] are two key secondary injuries that may induce subsequent neuronal cell death, including necrosis, autophagy or apoptosis [3, 4]. These injuries are associated with changes in certain parameters including glutamic acid transporter-1 (GLT-1) and the receptors NMDAR2A and NMDAR2B, microglia and astrocyte activation, and tumor necrosis factor α (TNF-α) production.

Thus, it is important to identify effective therapeutic drugs to overcome these secondary injuries. However, the development of new pharmacological drugs is a long and challenging process. Therefore, currently, drug repurposing is an alternative choice for novel drug discovery [5].

Ceftriaxone, a β-lactam antibiotic, is a long used and safe treatment for central nervous system CNS infection at an antibacterial dosage of 50–100 mg/kg [6]. It is permeable to the blood–brain barrier, has clear pharmacokinetic activity in the CNS [7], and is generally well tolerated, with a low incidence of adverse effects [8, 9]. Therefore, ceftriaxone is a potential drug candidate for other CNS insults.

Currently, ceftriaxone has been demonstrated to have beneficial effects in experimental Alzheimer’s disease, Parkinson’s disease, Huntington’s disease, stroke, brain ischemia, seizure, drug/alcohol dependency and withdrawal [10] and TBI models [11–14]. It was thought to exert its neuroprotective effect by increasing the expression and activity of GLT-1 to decrease the synaptic excitatory glutamate concentration. However, the experimental dosage of 200–250 mg/kg was much higher than the usual clinical dosage of 50–100 mg/kg. Furthermore, its effect on the anti-neuroinflammatory-associated response is still unclear and warrants further study.

In the current study, we hypothesized that ceftriaxone may have neuroprotective effects and not by its antibacterial effects at different dosages. The aim of the present study was to determine the neuroprotective mechanism of ceftriaxone after TBI, especially the effects on intracranial pressure (ICP), GLT-1, glutamate receptors NMDAR2A (post-synaptic) and NMDAR2B (extra-synaptic), neuronal apoptosis, and neuroinflammation-associated effects (microglia and astrocyte activation and TNF-α expression), and to improve functional outcomes using the clinical dosage of 50 mg/kg and an experimental dosage of 250 mg/kg.

## Methods

### Experimental design

**Fig. 1:**
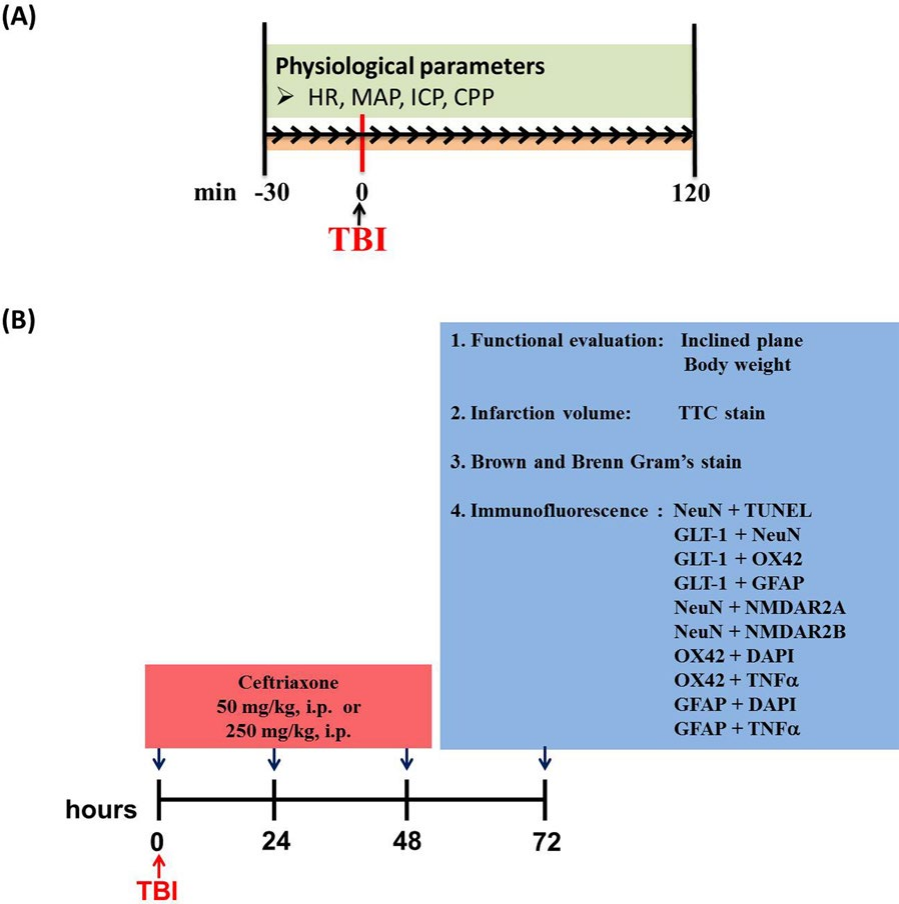
The overall experimental protocol in the current study

As shown in Fig. 1, in the first part, we tested the effects of ceftriaxone on physiological parameters. We continuously measured the mean arterial pressure (MAP), ICP, cerebral perfusion pressure (CPP) and heart rate for 120 min after TBI was induced. In the second part, ceftriaxone (50 mg/kg or 250 mg/kg) was given through the intraperitoneal route for 3 consecutive days. We attempted to elucidate whether these different dosages were associated with microglia and astrocyte activation, tumor necrosis factor α (TNF-α) expression, neuronal apoptosis, and cerebral injury in the ipsilateral injury side of the cortex using immunofluorescence staining and triphenyltetrazolium chloride (TTC) staining, while motor function was tested by the inclined plane at 72 h after TBI. The observational period set at 72 h after TBI was based on our previous study in TBI [15].

### Animals

Adult male Sprague–Dawley (SD) rats were obtained commercially from BioLASCO Taiwan Co., Ltd. and used in this study. The animals were kept under a 12/12-h light/dark cycle and allowed free access to food and water. All of the experimental procedures were approved by the Chi Mei Medical Center’s Animal Care and Use Committee and conformed to the NIH guidelines (Approval No. 10808602), including minimizing discomfort to the animals during surgery and during the recovery period. At the end of the experiments, the rats were euthanized by an overdose of urethane (intraperitoneal injections of 2 ml of 500 mg/ml urethane solution) until deep unconscious condition determined by the absence of visible breathing. All the animal experiments were carried out in compliance with the ARRIVE guidelines. To avoid potential interrater variability, all rats were measured by the same laboratory experimenter.

### Traumatic brain injury

Animals were anesthetized and intramuscularly injected with a mixture of ketamine (44 mg/kg, i.m.; Nankuang Pharmaceutical, Taiwan), atropine (0.063 mg/kg; Sintong Chemical Ind. Co., Taiwan), and Rompun (6.77 mg/kg, Bayer, Germany). Using a stereotaxic frame, a right craniectomy (with a radius of 2 mm) was performed 4 mm from the bregma and 3 mm from sagittal sutures. A fluid percussion device (VCU Biomedical Engineering, Richmond, VA, USA) was connected, and the brain was injured by rapidly injecting saline into the closed cranial cavity with a 2.2 atm plus 25 ms percussion. The detailed procedures are as described by Tsai et al. [15].

### Surgery and physiological parameter monitoring

The right femoral arteries of rats were cannulated with polyethylene tubing (PE50) for blood pressure monitoring. MAP and heart rate (HR) were monitored continuously with a pressure transducer after induction of TBI. An ICP microsensor (Codman and Shurtlef, Inc., Rayman, MA, USA) was placed in the parenchyma of the left frontal lobe of each rat. ICP was monitored continuously, and ICP and CPP values were recorded at 5-min intervals in the first 120 min after TBI. The CPP value was defined as MAP-ICP [16]. Colon temperatures were measured with an electronic thermometer (model 43 TE; YSI, Inc, Yellow Springs, OH) and a temperature probe (series 400; YSI, Inc).

### Treatment intervention

Anesthetized male SD rats were randomized and divided into sham-operated, TBI + vehicle, and TBI + ceftriaxone groups. Animals received three injections of ceftriaxone (50 mg/kg or 250 mg/kg) or vehicle intraperitoneally, including immediately, 24 h and 48 h after TBI. Vehicle for the TBI + vehicle group was normal saline (0.9%, Sintong, TAIWAN BIOTECH CO., LTD.), while the TBI + ceftriaxone-treated group received 50 mg/kg or 250 mg/kg ceftriaxone, dissolved in normal saline (Sintong TAIWAN BIOTECH CO., LTD.).

### Motor function test

The animals were placed perpendicular to the 20 × 20-cm buffer ribbed surface of an inclined plane. The maximal angle at which an animal could remain on the inclined plane was recorded. Motor deficit measurements were performed at 72 h after TBI.

### Cerebral injury volume assay

TTC staining was performed to measure the injury volume at 72 h following TBI [15]. The volume of injury, as revealed by negative TTC staining indicating dehydrogenase-deficient tissue, was measured in each slice and summed using computerized planimetry (PC-based Image Tools software, Image-Pro Plus Media Cybernetics, Inc., Rockville, MD, USA). The infarction volume was calculated as 2 mm (thickness of the slice) × [sum of the injury areas in all brain slices (mm2)] [17].

### Immunofluorescence assay

Seventy-two hours after TBI, adjacent 6-μm sections corresponding to coronal coordinates 0.20 mm to 0.70 mm anterior to bregma were incubated with primary antibodies in PBS containing 0.5% normal bovine serum overnight at 4 °C. After being washed in PBS, the sections were incubated with secondary antibodies for 1 h at room temperature. The number of immunofluorescent-positive cells was calculated in two coronal sections from each rat using computerized planimetry corresponding to the perilesioned cortical region (400× magnification, Image-Pro Plus Media Cybernetics, Inc. Washington Street, Rockville, USA), and the number of positive cells was expressed as the mean number of positive cells in the region of interest. The details of the antibodies used are summarized in Table 1.

**Table 1:** The detailed information of antibodies used in current study

Antibody	Source	Catalog number	Working dilution
TUNEL kit	Clontech(Palo Alto, CA)	630108	
Mouse anti-NeuN	Abcam(Cambridge, UK)	ab104224	1:600
Mouse anti-OX42	Abcam(Cambridge, UK)	ab1211	1:200
Mouse anti-GFAP	Cell signaling technology(Beverly, MA)	3670	1:800
Rabbit anti-GLT1	Abcam(Cambridge, UK)	ab41621	1:100
Rabbit anti-NMDAR2A	Chemicon international(Billerica, MA)	AB1555	1:600
Rabbit anti-NMDAR2B	Abcam(Cambridge, UK)	ab65783	1:500
Rabbit anti-TNFα	Abcam(Cambridge, UK)	ab6671	1:400

### Histological and pathological staining at 72 h after TBI

#### Paraffin sections

The wet tissues were trimmed and dehydrated with serial alcohol solution. Next, the tissues were embedded in paraffin wax. The paraffin-embedded tissues were cut at a thickness of 3—5 μm.

The slide-mounted paraffin sections -were deparaffinized and hydrated with xylene and serial alcohol solutions. One ml 1% crystal violet was mixed with 5 drops 5% sodium bicarbonate and then added to slides held in a staining rack. The slides were gently agitated to ensure that each section was covered, and then the sections were stained for 1 min. The slides were rinsed in distilled water, flooded with Lugol’siodine for 1 min and rinsed again with water. The sections were blotted dry and breathed on, and then acetone was quickly added to the section until the color stopped running off. The slides were washed in tap water. Slides were placed on a staining rack, and basic fuchsin was applied to the tissue sections and stained for 3 min. The slides were then washed in tap water and blotted gently to remove excess fluid but not to completely dry. The slides were then dipped rapidly into acetone followed by picric acid-acetone and then acetone-xylene.

Gram-positive bacteria were stained blue; gram-negative bacteria were stained red. Cell nuclei were stained red to yellow. Backgrounds were stained yellow. All glass slides were digitized with a MoticEasyscan Digital Slide Scanner (Motic Hong Kong Limited, Hong Kong, China) at × 40 (0.26 μm/pixel) with high precision (high precision autofocus). MoticEasyscan whole-slide images were viewed with DSAssistant and EasyScanner software at Li-Tzung Biotechnology Inc. (Kaohsiung, Taiwan).

### Statistical analysis

All of the data were analyzed using SigmaPlot, version 10.0 for Windows (Systat Software, San Jose, CA) or GraphPad Prism 6.0 (GraphPad Software, Inc., San Diego, CA, USA) in this study. The results are expressed as the mean with standard deviation of the mean for the experiments. Repeated measures analysis of variance (ANOVA) was conducted to test treatment-by-time interactions and the effect of treatment over time on each score. Two-way ANOVA followed by the post hoc Scheffe’s test was used to estimate the significant difference between these four groups. The box plots were used to present the distribution difference between four study groups. In addition, Wilcoxon ranked sum test was used to estimate the statistical difference. P-values < 0.05 were considered statistically significant.

## Results

### Basic data for the experimental rats

A total of 72 10-week-old male rats weighing 380 ± 22 g were used in the current study. The fluid percussion force was 2.20 ± 0.015 atm. The colonic temperature was controlled between 36 and 37 °C with lamp insulation during and up to 120 min after injury. No animal death or complications occurred over the course of the experiments.

### Part I: Physiological parameter evaluation in the first 120 min after TBI

#### Ceftriaxone-treated groups had significantly lower ICP during the initial 120 min after TBI

**Fig. 2:**
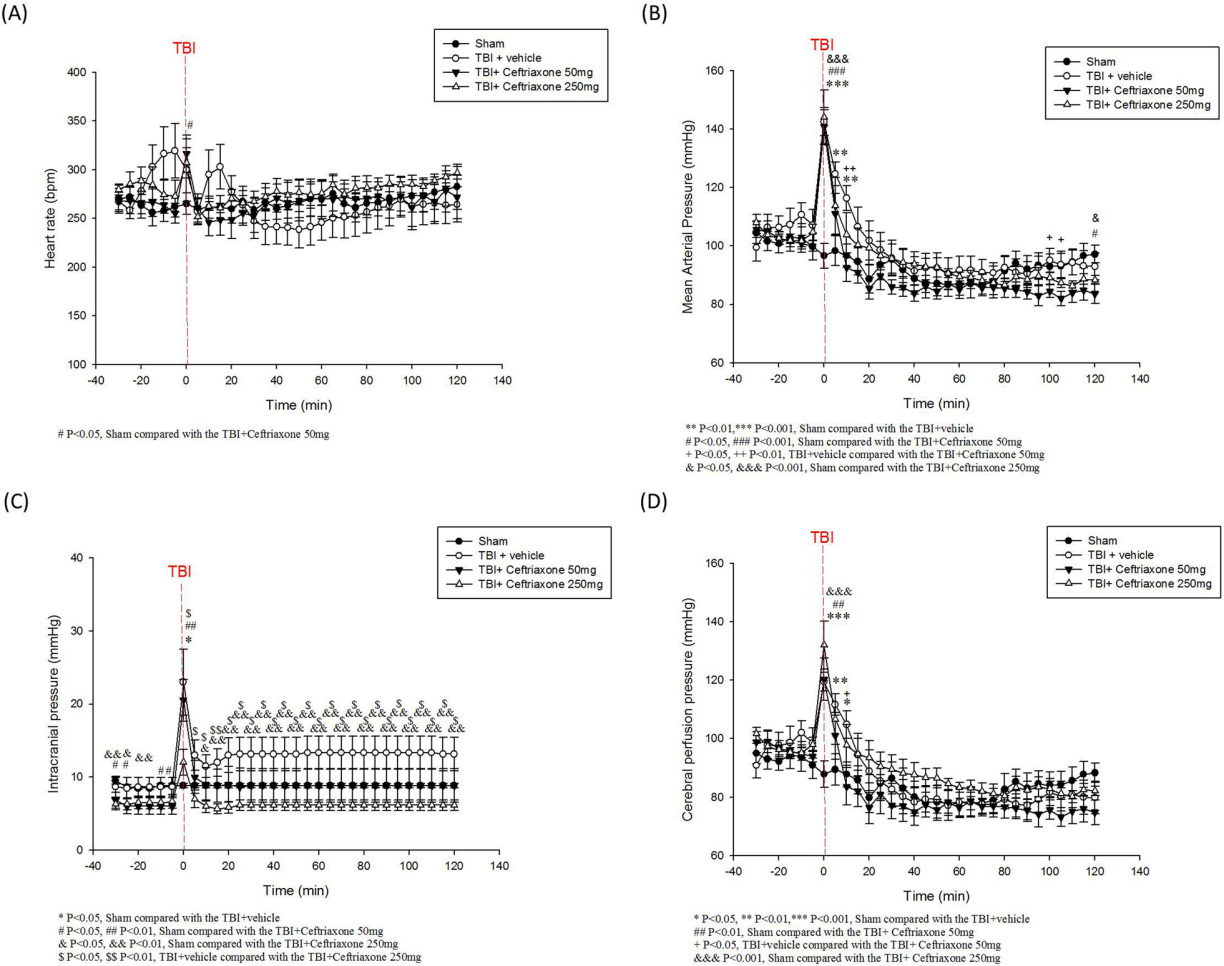
Effects of ceftriaxone on TBI-induced **a** heart rate (HR), **b** mean arterial pressure (MAP), **c** intracranial pressure (ICP) and **d** cerebral perfusion pressure (CPP) in the initial 120 min after traumatic brain injury (TBI). ***p < 0.001, compared with the sham group; ^#^p < 0.05, ^##^p < 0.01, compared with the TBI + vehicle group; ^$^p < 0.05, compared with the TBI + ceftriaxone 50 mg/kg group, n = 6 in each group

Since a high ICP could affect the functional outcome [18], we first tested the effects of ceftriaxone on physiological parameters, including HR, MAP, ICP and CPP, during the initial 120 min after TBI. In the TBI + vehicle group, the ICPs were higher at 0–120 min after the start of TBI than they were for sham-operated controls. In contrast, the values for ICP in the TBI + ceftriaxone (250 mg/kg/day) group were significantly lower than those of the sham-operated control and vehicle + TBI groups, ^*^p < 0.05, n = 6 in each group. Simultaneously, the TBI + ceftriaxone (250 mg/kg/d) group had a trend of higher CPP during the initial 120 min after TBI (Fig. 2).

### Part II: Functional outcome and neuropathology investigation at 72 h after TBI

#### Ceftriaxone significantly improved body weight loss at 72 h after TBI

**Fig. 3:**
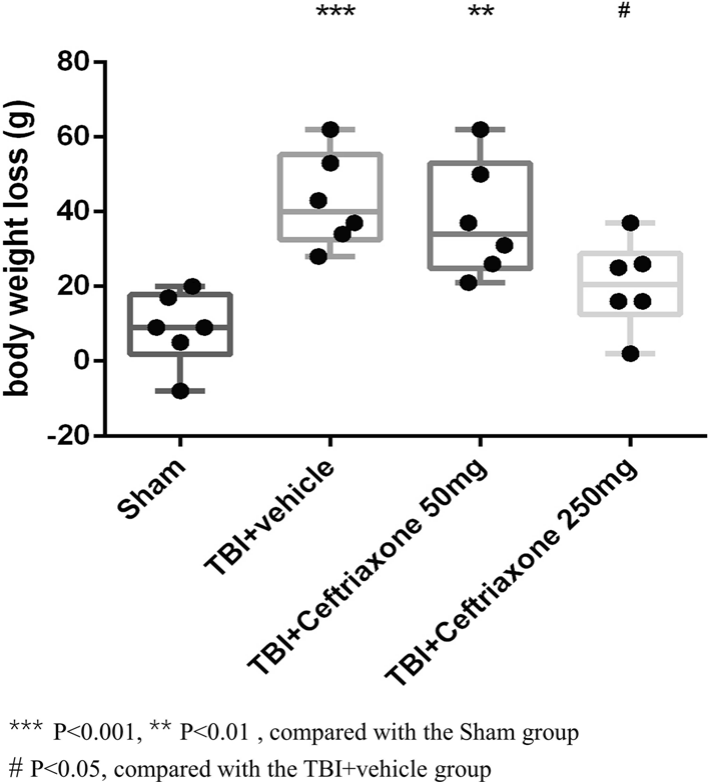
Effects of TBI-induced body weight losses at 72 h after TBI. ***p < 0.001, **p < 0.05 compared with the sham group; ^#^p < 0.05, compared with the TBI + vehicle group; n = 6 in each group

TBI in rats was characterized by body weight loss [19], and this correlated with injury severity [20]; therefore, we examined the ceftriaxone effect on body weight loss after TBI. The definition of body weight loss is the difference in body weight between 0 and 72 h after TBI. Figure 3 shows that TBI + vehicle rats had greater weight loss than sham-operated controls (^***^p < 0.001, n = 6 in each group). However, treatment with 250 mg/kg ceftriaxone after TBI significantly reversed the TBI-induced body weight loss (^#^p < 0.05, n = 6 in each group).

### Ceftriaxone significantly improved motor dysfunction at 72 h after TBI

**Fig. 4:**
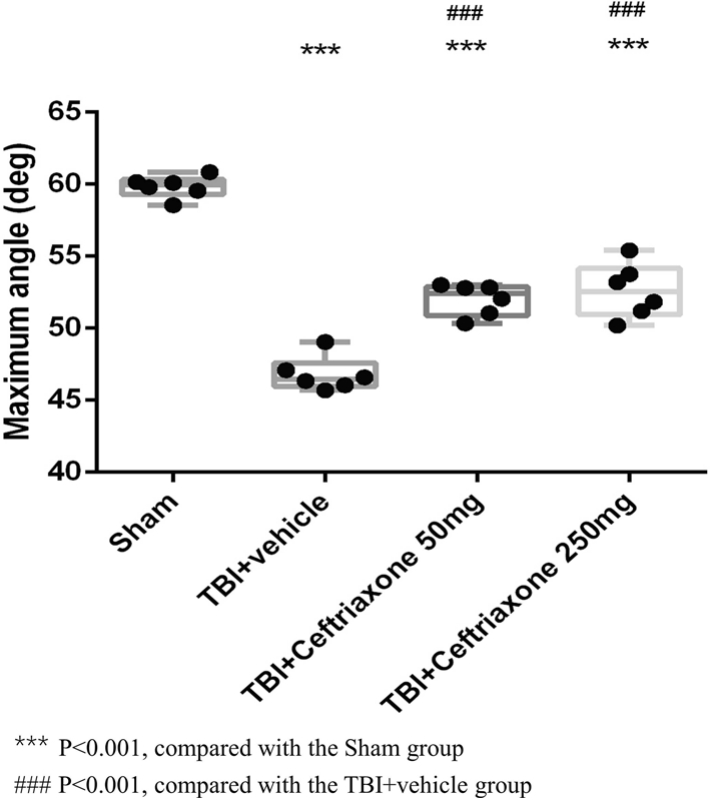
Effects of TBI-induced motor deficits at 72 h after TBI. ***p < 0.001, compared with the sham group; ^###^p < 0.001, compared with the TBI + vehicle group; n = 6 in each group

Then, we tested whether ceftriaxone has beneficial effects on functional outcomes such as motor dysfunction after TBI. At 72 h after TBI, the maximal grip angle in inclined plane tests showed that vehicle-treated TBI rats had significantly lower motor performance than sham-operated controls (^***^p < 0. 001, Fig. 4). TBI-induced motor dysfunction was significantly attenuated by both 50 mg/kg and 250 mg/kg ceftriaxone treatment (n = 6 in each group, Fig. 4).

### Ceftriaxone significantly decreased injury volume at 72 h after TBI

**Fig. 5:**
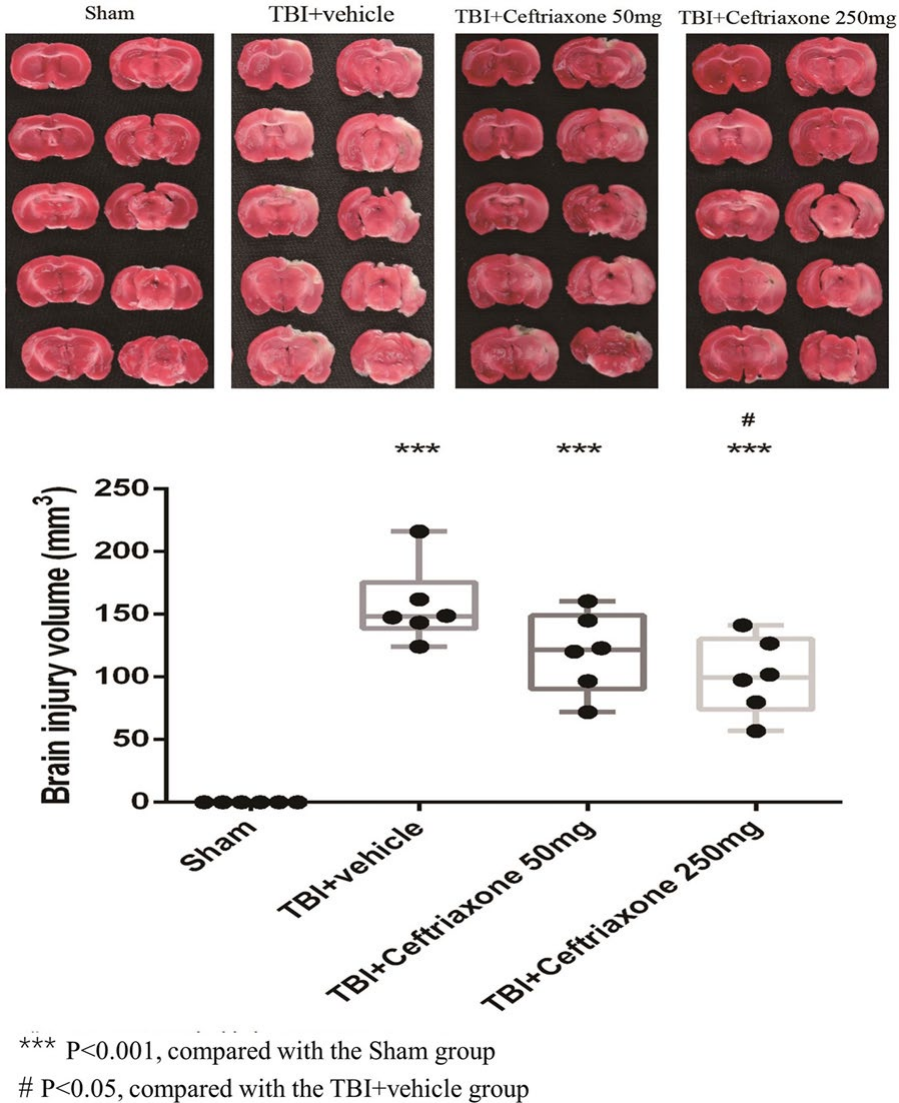
Effects of TBI-induced injury volumes in the perilesioned cortex at 72 h after TBI. ***p < 0.001, compared with the sham group; ^#^p < 0.05, compared with the TBI + vehicle group; n = 6 in each group

We examined whether the functional outcome improvement described above may relate to a decrease in injury volume after ceftriaxone treatment. At 72 h after TBI, the TTC-stained sections showed a significant increase in the infarct volume of the vehicle-treated TBI compared with those of sham TBI controls, (^***^p < 0.001, Fig. 5). The TBI-induced injury volume was significantly reversed by TBI treatment with 250 mg/kg ceftriaxone (^#^p < 0.05, n = 6 in each group, Fig. 5).

### Ceftriaxone significantly decreased neuronal apoptosis at 72 h after TBI

**Fig. 6:**
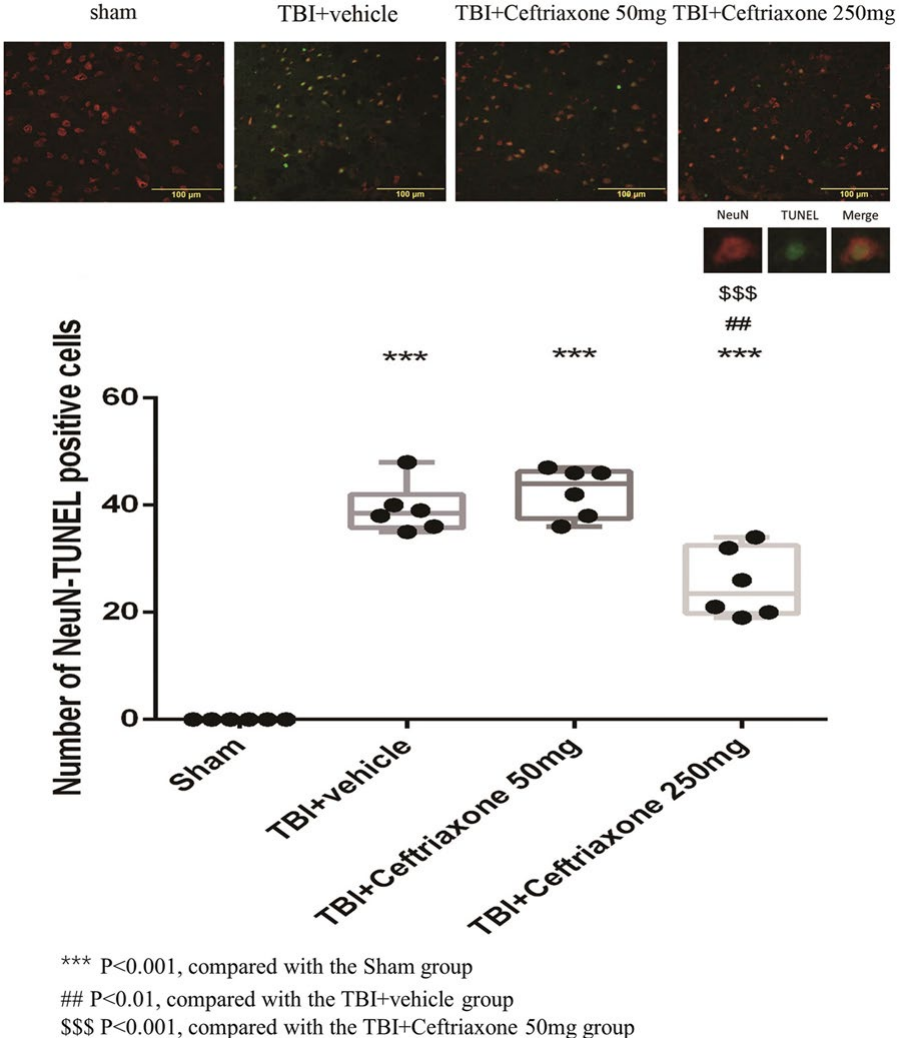
Effects of TBI-induced neuronal apoptosis in the perilesioned cortex at 72 h after TBI (markers NeuN plus TUNEL). The top panels depict representative NeuN plus TUNEL positive staining for one sham rat, one TBI + vehicle rat, one TBI + ceftriaxone (50 mg/kg)-treated rat, and one TBI + ceftriaxone (250 mg/kg)-treated rat. ***p < 0.001, compared with the sham group; ^##^p < 0.01, compared with the TBI + vehicle group; ^$$$^p < 0.001, compared with the TBI + ceftriaxone 50 mg/kg group, n = 6 in each group

Based on the results of the infarct volume, we next evaluated the effect of ceftriaxone on TBI-induced neuronal apoptosis. At 72 h after TBI, vehicle-treated TBI rats had significantly higher numbers of NeuN plus TUNEL-positive cells in the perilesioned cortex than sham controls (^***^p < 0.001, Fig. 6). TBI-induced neuronal apoptosis was significantly decreased by TBI treatment with 250 mg/kg ceftriaxone (^$$$^p < 0.001, Fig. 6), n = 6 in each group.

### Antibacterial effects of ceftriaxone at 72 h after TBI

**Fig. 7:**
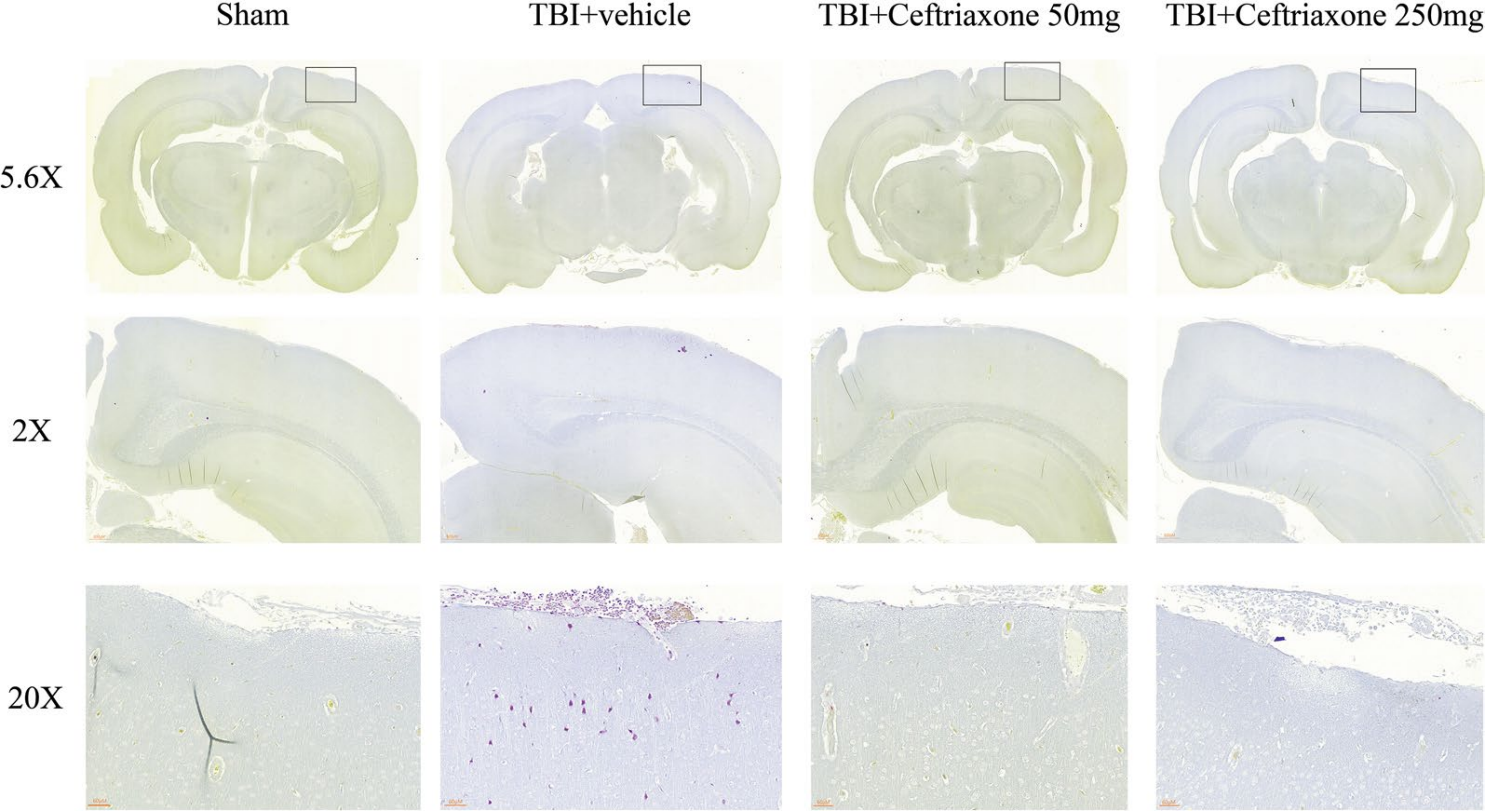
Histopathological changes at 72 h after TBI with Brown and Brenn’s Gram’s staining (5.6×, 20× and 200× magnification), n = 6 in each group

Next, we tested whether the neuroprotective effects of ceftriaxone were via antibacterial actions. Based on the morphology and staining, Fig. 7 shows that no obvious bacteria were found in sham-operated controls, TBI + vehicle and TBI + ceftriaxone (50 mg/kg or 250 mg/kg) rats.

### Ceftriaxone further significantly increased GLT-1 expression in neurons and microglia but not astrocyte expression at 72 h after TBI

**Fig. 8:**
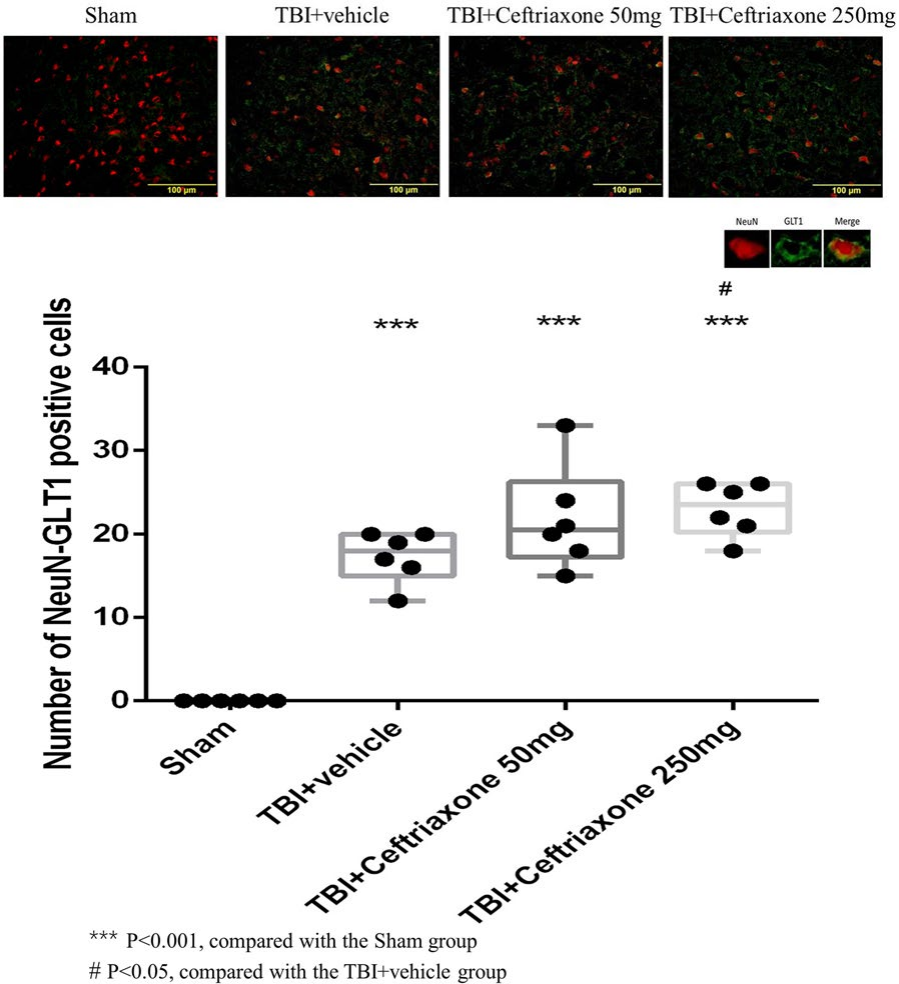
Effects of TBI-induced neuronal GLT-1 in the perilesioned cortex at 72 h after TBI (markers NeuN plus GLT-1). ***p < 0.001, compared with the sham group; ^#^p < 0.05, compared with the TBI + vehicle group; n = 6 in each group

**Fig. 9:**
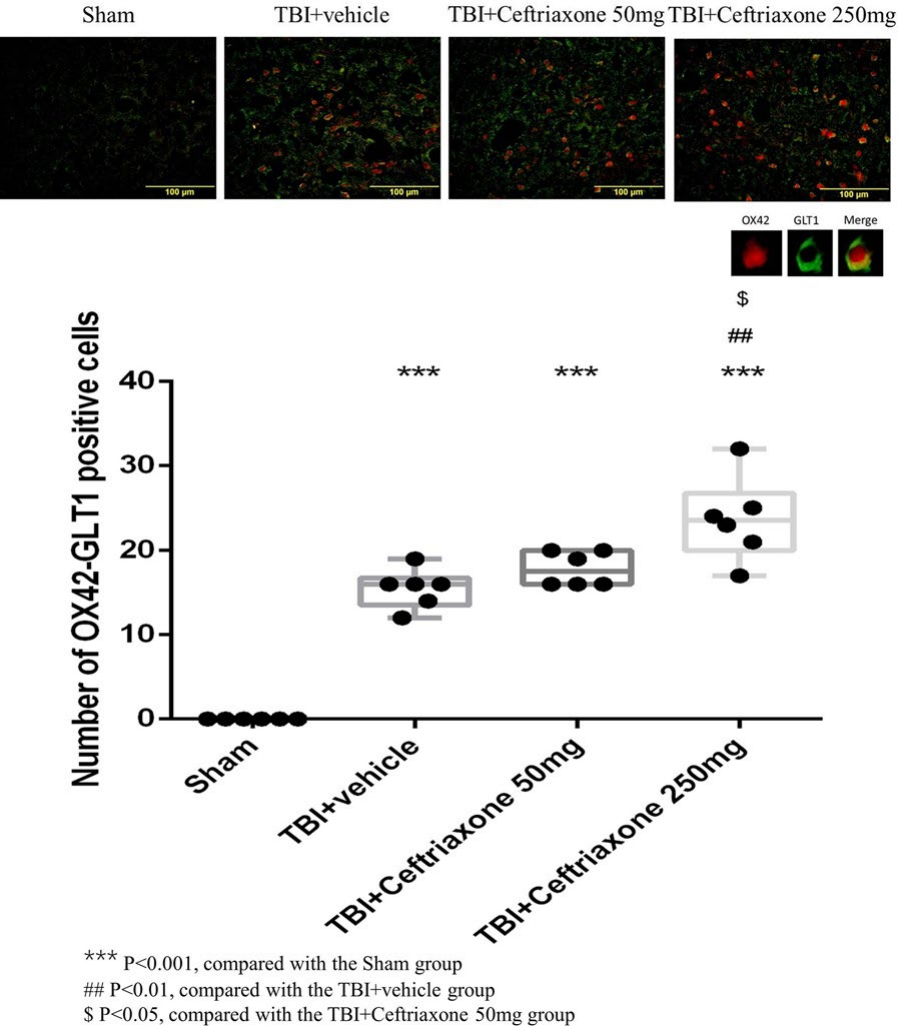
Effects of TBI-induced microglial GLT-1 in the perilesioned cortex at 72 h after TBI (markers OX42 plus GLT-1). ***p < 0.001, compared with the sham group; ^#^p < 0.05, ^##^p < 0.01, compared with the TBI + vehicle group; ***p < 0.001, compared with the sham group; ^$^p < 0.05, compared with the TBI + ceftriaxone 50 mg/kg group, n = 6 in each group

**Fig. 10:**
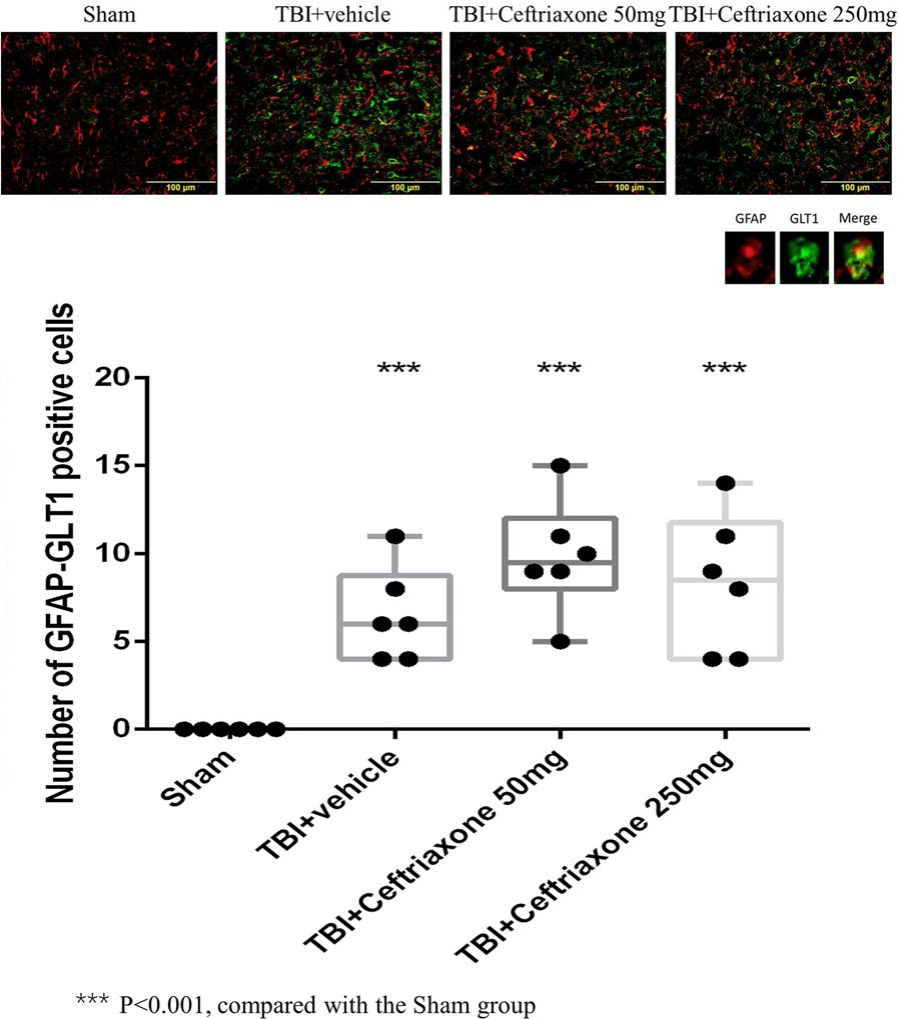
Effects of TBI-induced astroglial GLT-1 in the perilesioned cortex at 72 h after TBI (markers GFAP plus GLT-1). ***p < 0.001, compared with the sham group; #p < 0.05, compared with the TBI + vehicle group; ***p < 0.001, compared with the sham group, n = 6 in each group

To clarify GLT-1 expression in specific cells in vivo after different treatments, we next tested the effects of ceftriaxone on GLT-1 expression in astrocytes, microglia and neurons using double immunofluorescence staining. As shown in Figs. 8, 9, and 10, the number of GLT-1-positive neurons, microglia and astrocytes in the perilesioned cortex was significantly increased compared with that in sham rats at 72 h after TBI. The number of GLT-1-positive neurons and microglia in the perilesioned cortex after TBI was significantly increased further by TBI treatment with 250 mg/kg ceftriaxone. However, GLT-1 expression in astrocytes was not significantly increased after ceftriaxone treatment.

### Ceftriaxone significantly decreased NMDAR2B expression after TBI

**Fig. 11:**
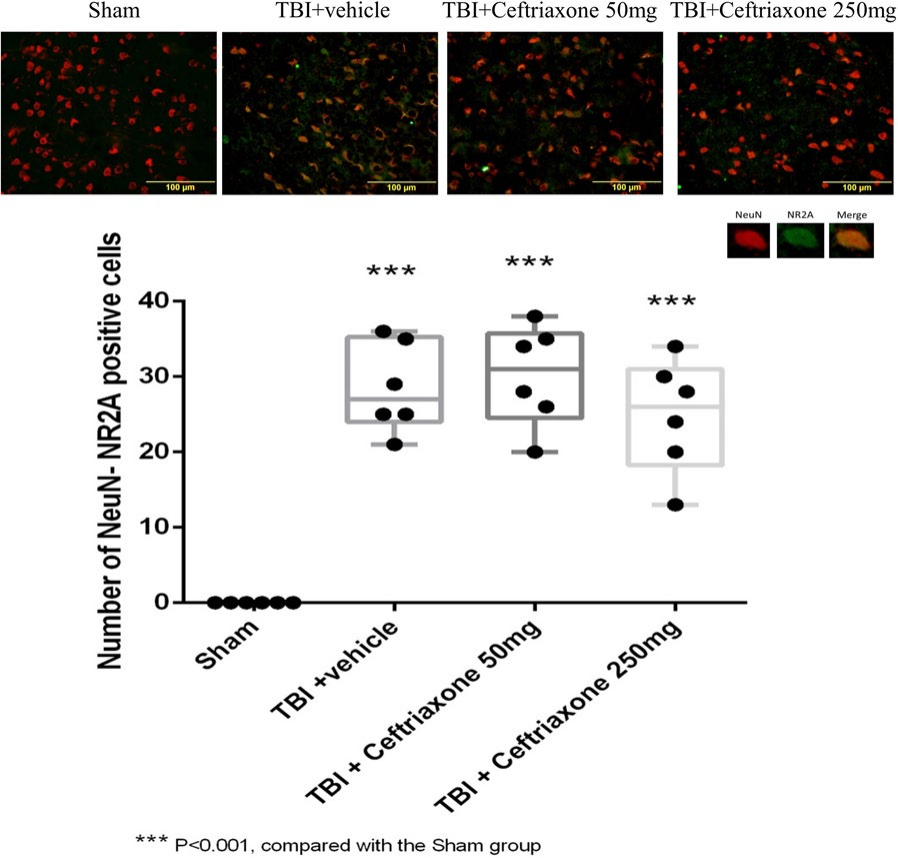
Effects of TBI-induced glutamate receptor subunits NMDAR2A at 72 h after TBI (marker Neu-N plus NMDAR2A). ***P < 0.001, compared with the Sham group, n = 6 in each group

**Fig. 12:**
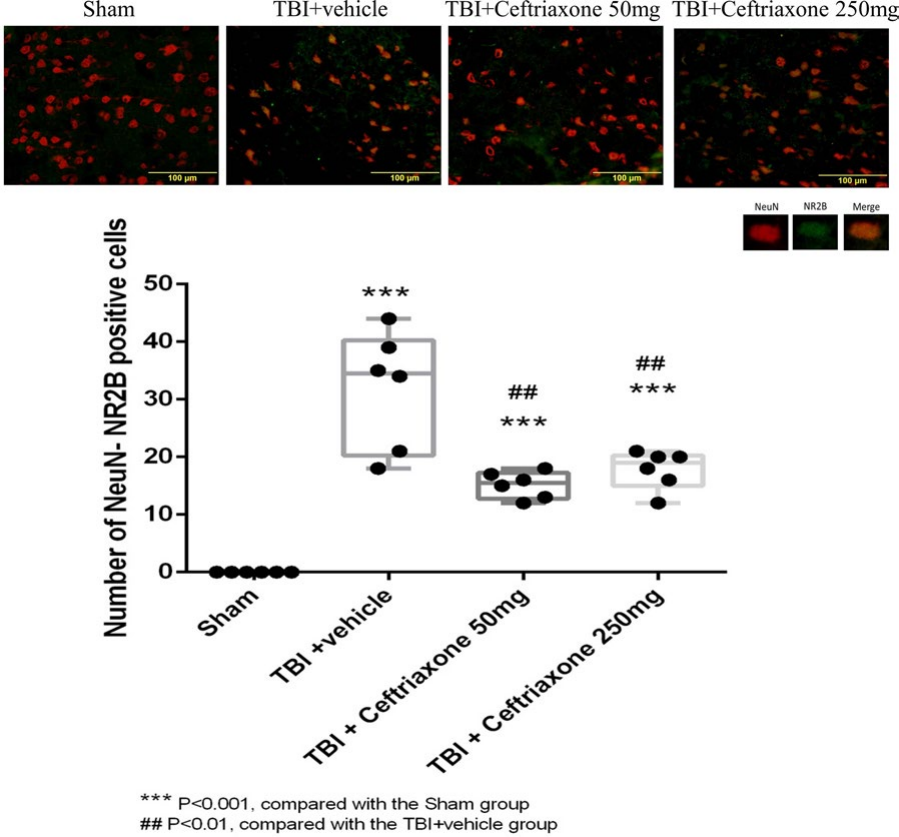
Effects of TBI-induced glutamate receptor subunits NMDAR2B at 72 h after TBI (marker Neu-N plus NMDAR2B). ***P < 0.001, compared with the Sham group, ^##^P < 0.01, compared with the TBI + vehicle group, n = 6 in each group

Next, we examined the effects of ceftriaxone after TBI on the expression of the postsynaptic neuroprotective NMDAR2A subunit and the extra-synaptic neurodestructive NMDAR2B subunit. In the NMDAR2A plus NeuN staining assay, the number of positive neuronal NMDAR2A cells in the perilesioned cortex of vehicle-treated TBI rats (28 ± 2.44) was significantly increased compared with that in the sham controls (0 ± 0, ^***^p < 0.001, n = 6 in each group, Fig. 11). However, the TBI-induced increase in neuronal NMDAR2A-positive cells was not significantly changed by either 50 mg/kg or 250 mg/kg ceftriaxone therapy (n = 6 in each group, Table 2). When compared with the TBI + vehicle rats, the number of TBI-induced NMDAR2B plus NeuN stained cells was significantly decreased both at 50 mg/kg and 250 mg/kg in the TBI + ceftriaxone rats (n = 6 in each group, Fig. 12).

**Table 2:** Studies of Ceftriaxone effects on traumatic brain injury animal models

Parameters/Authors	Species weight	Injury model severity	Dosage and course	Region observed	Parameters observed	Results
Wei et al. 2012	SD Rat 180–220 g	Weight-drop 20 g/30 cm height	200 mg/kg Single dose	Cortex	Brain edema TNF-α IL-6 IL-1β INF-r GLT-1 Learning and memory	Increase expression of GLT-1 Improving cognitive function Alleviating brain edema Reducing excitotoxicity and neuroinflammation
Cui et al. 2013	SD rat 300–330 g	Weight-drop 450 g/1.5 m height	200 mg/kg/d × 5 days	Hippocampus	Brain edema Autophage GLT-1 Learning and memory	Upper regulation of GLT-1 Suppression neuronal autophage Reducing brain edema
Goodrich et al. 2013	Long-Evans 367 ± 36 g	Fluid percussion 2.3 ± 0.1 atm	200 mg/kg/d × 7 days	Cortex	GLT-1 GFAP Seizure	Increase GLT-1 protein expression Decrease GFAP expression Reduce seizure duration
Hameed et al. 2019	SD rat 367 ± 36 g	Fluid percussion 2.3 ± 0.1 atm	250 mg/kg/d × 7 days	Cortex	Interneuron GLT-1 GABA	Prevent cortical inhibitory interneuron dysfunction Preserving GLT-1 expression
Present study 2020	SD rat 380 ± 22 g	Fluid percussion 2.20 ± 0.01 atm	50 mg/ kg/d or 250 mg/kg/d × 3 days	Cortex	TTC Motor TTC TUNEL GLT-1 OX42,TNF-α ICP	Increase GLT-1 expression in microglia & neuron Improved motor dysfunction Decrease infarct volume, apoptosis Attenuated neuroinflammation Lower ICP in initial 120 min after TBI

SD, Sprague–Dawley; FPI, fluid percussion injury

### Ceftriaxone significantly decreased TBI-induced microglial activation and TNF-α expression in microglia in the perilesioned cortex at 72 h after TBI

**Fig. 13:**
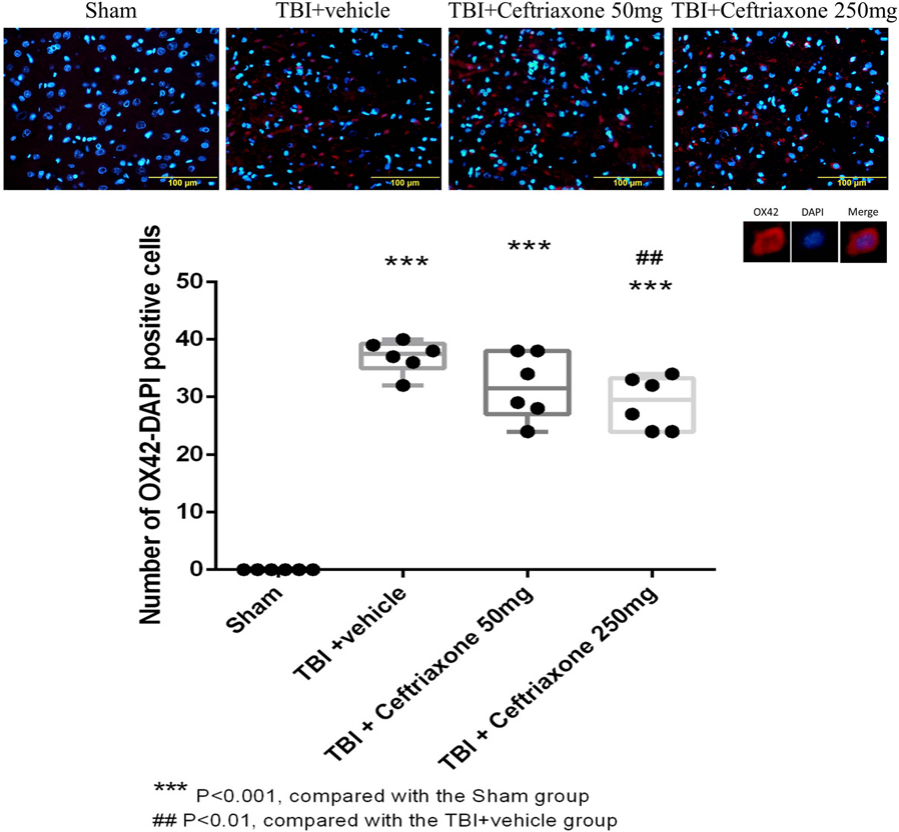
Effects of TBI-induced microglial expression in the perilesioned cortex at 72 h after TBI (markers OX42 plus DAPI). ***p < 0.001, compared with the sham group; ^##^p < 0.01, compared with the TBI + vehicle group; n = 6 in each group

Microglia is the major source of TNF-α production after TBI [21]; therefore, we examined whether the neuroprotective effect of ceftriaxone was anti-inflammatory. The OX42 plus 4',6-diamidino-2-phenylindole (DAPI) double staining showed that the number of positive cells in the perilesioned cortex of the vehicle-treated TBI rats (37 ± 1.15) was significantly higher than that in the sham-operated rats (0 ± 0, ^***^P < 0.001). Furthermore, TBI-induced positive OX42 in the DAPI-positive cells was significantly lower in the 250 mg/kg ceftriaxone treatment group compared with the TBI vehicle-treated rats (29 ± 18.6, ^##^P < 0.01, Fig. 13).

**Fig. 14:**
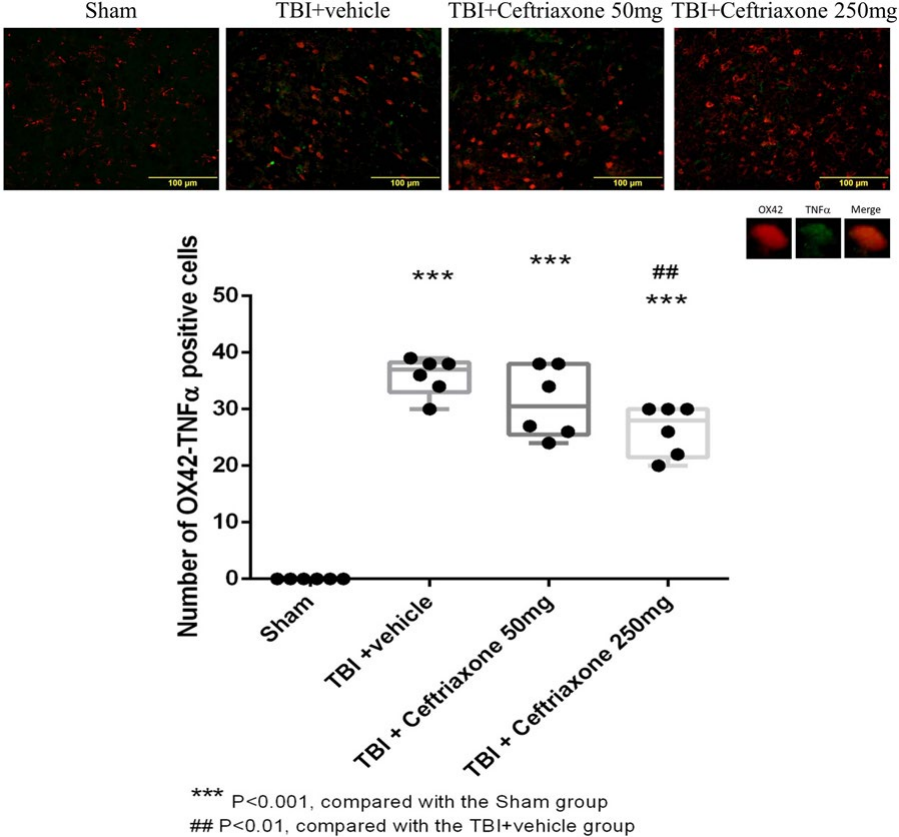
Effects of TBI-induced TNF-α expression in the microglia in the perilesioned cortex at 72 h after TBI (markers OX42 plus TNF-α). ***p < 0.001, compared with the sham group; ^##^p < 0.01, compared with the TBI + vehicle group; n = 6 in each group

Simultaneously, OX42 plus TNF-α double staining in the perilesioned cortex of the vehicle-treated TBI rats (35 ± 1.37) was significantly higher than that in the sham-operated rats (0 ± 0, ^***^P < 0.001). The TBI-induced increase was significantly reversed by TBI treatment with 250 mg/kg ceftriaxone (26 ± 1.82, ^##^p < 0.01, n = 6 in each group, Fig. 14). These results support that antineuroinflammation is one mechanism for the neuroprotective effects of ceftriaxone.

### Ceftriaxone significantly decreased TBI-induced astrocytic activation but not the TNF-α expression in astrocyte in the perilesioned cortex at 72 h after TBI

**Fig. 15:**
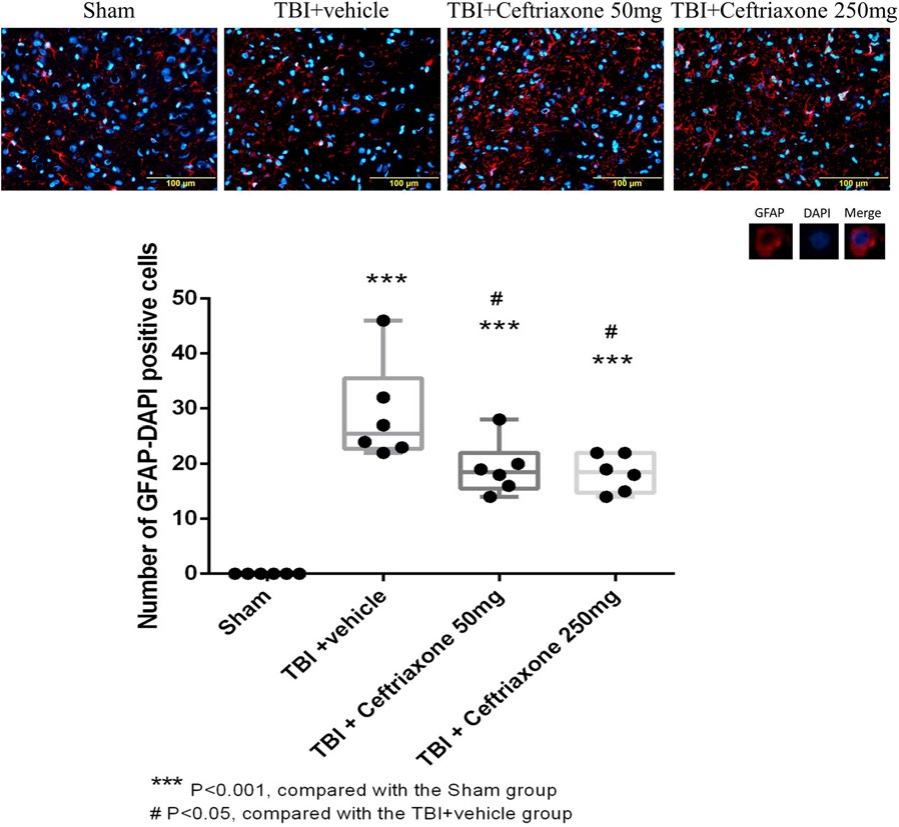
Effects of TBI-induced astrocytic expression in the perilesioned cortex at 72 h after TBI (markers GFAP plus DAPI). ***p < 0.001, compared with the sham group; ^#^p < 0.05, compared with the TBI + vehicle group; n = 6 in each group

**Fig. 16:**
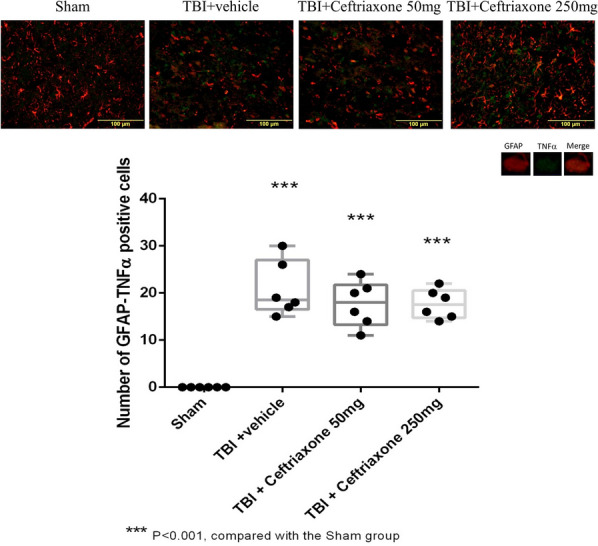
Effects of TBI-induced TNF-α expression in the astrocyte in the perilesioned cortex at 72 h after TBI (markers NeuN plus GLT-1). ***p < 0.001, compared with the sham group, n = 6 in each group

Similar to the microglial reaction,, TBI-induced positive GFAP in the DAPI-positive cells was significantly lower both in the 250 mg/kg and 50 mg/Kg ceftriaxone treatment group compared with the TBI vehicle-treated rats (n = 6 in each group, Fig. 15). However, the TBI-induced positive TNF-α in the GFAP-positive cells was not reversed by TBI treatment with 50 or 250 mg/kg ceftriaxone (Fig. 16).

## Discussion

### Novelty of the current study

According to our review of the literature, this study is the first to demonstrate the effects of different dosages of ceftriaxone on TBI-induced glutamate receptor expression and neuroinflammation. We provide some new information on ceftriaxone in the field of neurocritical care, including that (1) the mechanism of neuroprotection of ceftriaxone is not related to its antibacterial effect; (2) treatment with 250 mg/kg/day ceftriaxone for 3 days may increase GLT-1 expression, decrease neuroinflammation injury volume and neuronal apoptosis, and improve motor dysfunction and body weight loss; and (3) treatment with 50 mg/kg/day or 250 mg/kg/day ceftriaxone for 3 days may attenuate extrasynaptic neurodestructive NMDAR2B subunit expression.

### Determination of the dosage and time course of ceftriaxone administration

In the current study, the dosage of ceftriaxone administration (250 mg/kg/day) was in accordance with the Hameed et al. TBI animal model [14]. The administration time course immediately, 24 h and 48 h after TBI was based on the fact that TBI-induced cell apoptosis was maximal at 2–3 days after TBI [22] and significantly increased at 72 h after TBI compared with that of the sham group [15]. Based on our previous study, the observation end-point 72 h post-injury TBI were selected because lateral fluid percussion caused body weight loss, neuroinflammation and apoptosis compared with sham group in this time point [15].

Compared with the usual daily dose of ceftriaxone, which is 1–2 g administered once daily in adults and 50–100 mg/kg once daily in pediatric patients [6], the dose used in this study (250 mg/kg per day) is relatively high. However, in a preclinical study, the no-observed-adverse-effect dose level was 500 mg/kg/day in both sexes associated with the long-term administration of ceftriaxone for 6 months [23]. Therefore, the dosage and time course of ceftriaxone administration in our study may have potential for clinical applications in the future.

### Early effects of ceftriaxone on physiological parameters during the initial 120 min after TBI

Consistent with previous studies [24, 25], in the first 10 min, our results showed an abrupt increase in ICP after TBI followed by a gradual decrease, including in the TBI-vehicle, low-dose and high-dose ceftriaxone treatment groups. The abrupt increase in ICP may be due to the sudden injection of a small amount of fluid into the cranial cavity due to the impact force, which then displaces or distorts the cerebral vessels, followed by an immediate decrease in cerebral blood flow and ultimately inadequate cerebral perfusion [24, 26]. We suggest that the gradual decrease in ICP within minutes after the peak may be related to cerebral hyperperfusion and effective autoregulation after TBI [26]. An undetected CSF leakage occurring at the ICP catheter insertion may be another cause of reduced ICP [24]. These phenomena were not associated with ceftriaxone treatment.

Compared to the TBI groups in our study, the ceftriaxone-treated group (250 mg/kg, i.p.) had a significantly lower ICP, mostly occurring approximately 10 min during the initial 120 min after TBI. We consider that these early beneficial effects of ceftriaxone on ICP may mitigate the development of secondary injury, such as neuroinflammation and excitotoxicity, and ultimately improve outcome [18] on the 3rd day after TBI according to our study. However, we did not measure whether ICP was altered as a result when ceftriaxone was treated alone in the absence of TBI. This issue needs clarified further.

Yuk et al. demonstrated that following a single intravenous injection of 2 gm ceftriaxone, the peak concentration of ceftriaxone was detected at 30 min post treatment [27]. However, we did not investigate the relationship between the brain ceftriaxone concentration and physiological parameter changes after TBI. This relationship warrants further investigation.

Based on the fact that increased ICP leads to increases in sympathetic activity, which is responsible for changes in gastrointestinal motility, water and electrolyte absorption [28], the relationship between ceftriaxone and gastrointestinal function warrants further investigation.

### Ceftriaxone effects on body weight loss at 72 h after TBI

TBI rats are characterized by body weight loss [19], which is correlated with injury severity [20, 29]. The causes of body weight loss after TBI may relate to anorexia, enhancement of proteolysis, and distal intestinal atrophy [30] and affect gut microbial diversity [31, 32].

In our previous study, we found that TBI increased local and systemic TNF expression [33]. In the current study, ceftriaxone significantly decreased microglial activation and TNF-α expression in microglia after TBI. Since disruption of the brain-gut axis may lead to dysfunction of the gastrointestinal system [34], whether ceftriaxone may have beneficial effects on the gastrointestinal system, especially TNF-α expression in gut, and finally decrease the body weight loss after TBI is worthy of future investigation.

### Ceftriaxone effects on motor deficiency after TBI

Following TBI, the mechanism of cell death includes necrosis, apoptosis and autophagy, which may affect functional outcomes [4, 35]. Cui et al. showed that treatment with ceftriaxone at 200 mg/kg/day for 5 days attenuated neuronal autophagy in the hippocampus after TBI12. In the current study, we added the new information that treatment with 250 mg/kg/day ceftriaxone for 3 days attenuated neuronal apoptosis in the perilesioned cortex and synergistically improved motor deficiency. However, treatment with 50 mg/kg/day ceftriaxone for 3 days did not attenuated neuronal apoptosis but improved motor dysfunction. Therefore, we speculate that neuronal autophagy may be involved in our model in higher dose of ceftriaxone treatment, but the mechanisms need to be clarified. Furthermore, the effect of ceftriaxone on the cell necrosis after TBI also needs to be investigated in the future.

### Ceftriaxone effects on glutamate transporters at 72 h after TBI

GLT-1 is the major glutamate transporter responsible for glutamate uptake to decrease the accumulation of glutamate in the synaptic cleft and to reduce postsynaptic glutamate receptor overactivation [36]. GLT-1 is predominantly expressed in astroglia [36, 37] but is also present in neurons and in axon terminals [38]. Under physiological conditions, glutamate in the synaptic cleft is primarily transported by the glutamate transporter GLT-1 to astrocytes, where it is subsequently metabolized to glutamine and then safely transported back to neurons as glutamine.

However, under pathological conditions, the astrocyte glutamate transporter is downregulated and the microglia transporter is induced for glutamate clearance [39].

Our immunofluorescence assay results at 72 h after TBI show that neuronal GLT-1 levels were acutely elevated in the perilesioned cortex compared with sham rats and it is reasonable to believe that this is a protective effect to reduce the accumulation of glutamate in the synaptic cleft. Our results were consistent with Goodrich et al. in that they demonstrated that ceftriaxone treatment after TBI restored the expression of GLT-1 according to western blotting [13]. We further identified that treatment with 250 mg/kg ceftriaxone for 3 days after TBI further increased neuronal and microglial GLT-1 transporter but not astrocytic GLT1 transporter expression in the perilesioned cortex.

The possible reasons for the high expression of GLT-1 in microglia in the injured tissue may be related to the proliferation of microglia there [40] and up-regulated GLT-1 in microglia to transport glutamate from the extracellular space for glumate clearance [39]. The low expression in astrocytes may be due to the accumulation of glutamate in astrocyte vesicles that retards transportation and the inability of GLT-1 to reform the sealed cell membrane structure [41]. However, these speculative hypotheses require further experiments beyond the scope of this report.

### Ceftriaxone effects on glutamate receptor subunits NMDAR2A and NMDAR2B at 72 h after TBI

Our results showed that treatment with 50 mg/kg/day or 250 mg/kg/day ceftriaxone for 3 days attenuated neurodestructive extrasynaptic NMDAR2B subunit expression. We speculate the decrease in NMDAR2B expression may be related to ceftriaxone increasing GLT-1 activity leading to the decreased presence of glutamic acid in the synaptic cleft and extrasynaptic region. However, the analysis of glutamate concentration in the extracellular space using microdialysis should be clarified in the future.

### Ceftriaxone effects on neuroinflammation and apoptosis after TBI

Our study is consistent with Wei et al. who demonstrated that ceftriaxone administration, using a single dose of ceftriaxone (200 mg/kg i.p.) after weight-drop brain injury, can upregulate GLT-1 and reduce TNF-α expression [11]. We further contribute the new information that ceftriaxone up-regulated microglia GLT-1 expression and attenuated microglial activation and TNF-α expression in activated microglia and synergistically reduced neuronal apoptosis according to immunofluorescence staining in the perilesioned cortex. Therefore, we suggest that the clearance of extracellular glutamate by up-regulated microglia GLT-1 might synergistically reduce neuroinflammation. Since excitotoxicity and neuroinflammation play important roles in triggering and sustaining the neurodegenerative process after TBI and TNF-α is a link between excitotoxicity and neuroinflammation [42], we suggest that ceftriaxone administration in the acute stage may be a promising therapeutic strategy for critical care after TBI.

### Ceftriaxone effects on neurons, microglia and astrocytes after TBI and possible molecular mechanisms

Our results showed that after TBI ceftriaxone increased neuronal GLT1 expression and attenuated NMDAR2B expression and neuronal apoptosis. Ceftriaxone also increased microglial GLT1 expression, microglial activation and TNF-α production. However, ceftriaxone attenuated astrocyte activation, but did not significantly alter TNF-α production and GLT1 expression in astrocytes. These results suggested that dysregulation of GLT-1 in different neuroglial cells is critically involved in inducing neuronal death after TBI. In the molecular transcriptional level, ceftriaxone treatment induced upregulation of GLT1 and GLT1 isoforms in association with activation of the Akt-NFκB signaling pathway and enhancement of the GLT-1 promoter activity and protein levels [43]. Whether the expression of Akt-NFκB signaling pathway is different in neurons, microglia and astrocytes, resulting in different GLT1 expression after TBI or ceftriaxone treatment, deserves further investigation in the future.

### Comparison of our results with other experimental TBI models treated with ceftriaxone

Table 2 summarizes the previous and current studies in animal models of TBI treated with ceftriaxone. There have been several different administration dosages and course methods for ceftriaxone in the experimental TBI field, including 200 mg/kg/d1 [11–13] and 250 mg/kg/day [14], which were much higher than the usual clinical dosage of 50–100 mg/kg/day [6]. The time courses were variable from a single dose [11], or for 5 days 12 to 7 days [13, 14]. The differences in animal weight, injury models and severity, administration dosage and time course, and regional observations inevitably led to variable outcomes. Our results showed that TBI treatment with ceftriaxone at both 50 and 250 mg/kg/day for 3 days may improve motor deficits. Therefore, we propose that without its antibacterial effect, ceftriaxone may be a potential candidate for neuroprotection after TBI.

### Limitations of the current study

Several limitations in our study should be mentioned. First, we only investigated the effects of GLT-1 and NMDA receptor subunits without detecting glutamate receptors such as α-amino–3-hydroxy-5 methyl-4-isoxazole propionic acid (AMPA), kainate, and metabotropic receptors, which may influence neuroinflammation and excitotoxicity [44]. Second, we only evaluated the perilesioned cortex. Whether ceftriaxone has a beneficial effect on the hippocampus, which is related to learning and memory performance and depression development after TBI, requires clarification using hippocampal histology staining and behavioral tests such as the Morris water maze and forced swimming test [45]. Third, we only investigated the short-term postinjury pharmacological intervention effects of ceftriaxone, while a 3-day time period assessment is useful to assess neuroinflammation-associated parameters of TBI-induced secondary insults [15]. Therefore, the longer-term neurological outcome and adverse reactions of ceftriaxone could be assessed in follow-up studies despite Ratti E showing no adverse effects of ceftriaxone when administered at 500 mg/kg/day for 6 months [23]. Fourth, we initiate ceftriaxone treatment right after impact has very little clinical relevance. Delayed treatment such as 30 min or 1 h or others after TBI to evaluate the effects of ceftriaxone on TBI should be performed in the future. Fifth, we only used a single functional method, an inclined plane test to measure motor functions of the upper and lower limbs after neural damage in rats. However, this test is not sufficient to exclude any possibility of motor weakness; therefore, multiple tests for motor function should be used in the future. Finally, to reinforce the essential impact of the results it would be evaluated the expression of the key caspases (i.e. casp-3/8/9, Bcl-proteins), the level of key pro- & anti-inflammatory cytokines/chemokines in the future.

## Conclusions

In acute stage after TBI, the neuroprotective effect of ceftriaxone may relate to increase GLT-1 expression and reduce neuroinflammation and neuronal apoptosis resulting in an improvement in functional outcomes. This neuroprotective effect is not related to its antibacterial effects.

## Data Availability

The datasets used and/or analyzed during the current study available from the corresponding author on reasonable request.

## Abbreviations

TBI: Traumatic brain injury
SD: Sprague–Dawley
TTC: Triphenyltetrazolium chloride
TNUEN: Terminal deoxynucleotidyl transferase-mediated dUTP-biotin nick end labeling
GFAP: Glial fibrillary acidic protein
DAPA: 4', 6-Diamidino-2-phenylindole
HR: Heart rate
MAP: Mean arterial pressure
ICP: Intracranial pressure
CPP: Cerebral perfusion pressure
GLT-1: Glutamate transporter 1
TNF-α: Tumor necrosis factor α
NR 2A: *N*-Methyl-d-aspartate receptor 2A
NR2B: *N*-Methyl-d-aspartate receptor 2B
